# Specificity and mechanism of action of alpha-helical membrane-active peptides interacting with model and biological membranes by single-molecule force spectroscopy

**DOI:** 10.1038/srep29145

**Published:** 2016-07-01

**Authors:** Shiyu Sun, Guangxu Zhao, Yibing Huang, Mingjun Cai, Yuping Shan, Hongda Wang, Yuxin Chen

**Affiliations:** 1 Key Laboratory for Molecular Enzymology and Engineering of the Ministry of Education, Jilin University, Changchun 130012, China; 2 School of Life Sciences, Jilin University, Changchun 130012, China; 3 National Engineering Laboratory for AIDS Vaccine, Jilin University, Changchun 130012, China; 4 State Key Laboratory of Electroanalytical Chemistry, Changchun Institute of Applied Chemistry, Chinese Academy of Sciences, Changchun, Jilin 130022, China; 5 University of Chinese Academy of Sciences, Beijing 100049, P.R. China

## Abstract

In this study, to systematically investigate the targeting specificity of membrane-active peptides on different types of cell membranes, we evaluated the effects of peptides on different large unilamellar vesicles mimicking prokaryotic, normal eukaryotic, and cancer cell membranes by single-molecule force spectroscopy and spectrum technology. We revealed that cationic membrane-active peptides can exclusively target negatively charged prokaryotic and cancer cell model membranes rather than normal eukaryotic cell model membranes. Using *Acholeplasma laidlawii*, 3T3-L1, and HeLa cells to represent prokaryotic cells, normal eukaryotic cells, and cancer cells in atomic force microscopy experiments, respectively, we further studied that the single-molecule targeting interaction between peptides and biological membranes. Antimicrobial and anticancer activities of peptides exhibited strong correlations with the interaction probability determined by single-molecule force spectroscopy, which illustrates strong correlations of peptide biological activities and peptide hydrophobicity and charge. Peptide specificity significantly depends on the lipid compositions of different cell membranes, which validates the *de novo* design of peptide therapeutics against bacteria and cancers.

Membrane-active peptides (MAPs), including antibacterial peptides, antifungal peptides, and anticancer peptides, have been widely studied and are promising drug candidates to compensate conventional antibiotics and chemotherapeutics^1^ A number of factors believed to be important for biological activities of MAPs have been identified, including the presence of both hydrophobic and basic residues, an amphipathic nature that segregates basic and hydrophobic residues, and an inducible or preformed secondary structure (α-helical or β-sheet)^2^. In the previous study, based on the “carpet-like” mechanism of bacterial membrane lysis^3^ and the “barrel-stave” mechanism of interactions of pore-forming peptides with cell membranes^4^, Chen *et al.* proposed “membrane discrimination” model, that is, peptide biological activities against prokaryotic and eukaryotic cells depend on the difference in membrane composition^2^. Although several aforementioned mechanisms have been proposed, the exact targeting specificity mechanism of MAPs is still unclear, due to the complexity on structure and function of biological membranes^5^.

Cell membranes are large complexes that consist of vast lipids, polysaccharides and proteins^5^. Most prokaryotic cells generally contain a cell wall with thick peptidoglycan layer. Gram-positive bacteria are surrounded by a single highly negatively charged cell membrane enriched in phosphatidylglycerol (PG), and Gram-negative bacteria have two highly negatively charged membranes enriched in PG, i.e. outer membrane in cell wall and cytoplasmic membrane^6^. *Acholeplasma laidlawii* (*A. laidlawii*) is a simple, cell wall-less prokaryotic microorganism used in this study as an example of prokaryotic membrane system^7^. To the mammalian tissue cells, dense proteins are exposed on the cytoplasmic side of nucleated eukaryotic cell membranes^8^, which are enriched in the zwitterionic phospholipids (neutral net charge), phosphatidylethanolamine (PE), phosphatidylcholine (PC), sphingomyelin (SM), and cholesterol (Chol)^9^. Red blood cells are simple eukaryotic cells, whose outer leaflet of membranes is quite smooth without proteins protruding out of the cell surface and thus bridge the gap between model membranes (only lipid bilayer) and nucleated eukaryotic membranes^5^,^10^. Compared with normal eukaryotic cells, the membrane components of cancer cells have more negatively charged components exposed on the outer membrane leaflet of cancer cells such as phosphatidylserine (PS) (3–9% of total phospholipids of membranes)^11^. Since MAPs are positively charged, the electrostatic interaction between MAPs and different membranes could be the driving force and the inherent distinctions in the lipid composition of different types of cell membranes may provide a basis for the selectivity of MAPs.

Many techniques have been employed to study the interactions between peptides and cell membrane, e.g., scan electron microscopy, confocal fluorescence microscopy and differential scanning calorimetry^12^. However, due to the limited spatial resolution of the above methods, many details on the targeting interaction between single peptides and cell membranes remain largely unknown until now. Atomic force microscopy (AFM) has been a powerful tool in biological applications due to its combination of single-molecule resolution and physiological conditions^13^. Single-molecule force spectroscopy (SMFS) based on AFM is highly sensitive, and the corresponding force spectroscopy allows to detect forces as low as 10 pN^14^. Researchers have used AFM to investigate interactions between nanoparticles and living cell membranes^14^. In this study, we systematically investigated the targeting specificity of MAPs with different types of model and cell membranes using SMFS based on AFM and fluorescence spectroscopy. Different large unilamellar vesicles (LUVs) were used to mimic prokaryotic cell membranes (PC/PG), normal eukaryotic cell membranes (PC/Chol), and cancer cell membranes (PC/SM/PE/PS/Chol) to understand the targeting mechanism of action of MAPs on the different model membranes using SMFS and spectrum spectroscopy. In addition, *A. laidlawii*, 3T3-L1 and HeLa cells were used to represent prokaryotic cells, normal eukaryotic cells and cancer cells, respectively, to study the mechanisms of interaction of MAPs with various biological cell membranes. Furthermore, Chicken erythrocyte cells were used to study the gap between model membranes and other biological cell membranes. We believe that peptide targeting specificity significantly depends on the lipid compositions of different cell membranes, which plays an important role on *de novo* design of peptide therapeutics against bacteria and cancers.

## Results

### Biological activities

The peptide design and biophysical properties are shown in the supporting information (Supplementary Fig. S1, Tables S1–S2). The MHC was determined by hRBCs to evaluate the cytotoxicity of MAPs against human normal cells (Table 1). It is clear that two peptides (V13L and A12L/A20L) with strong hydrophobicity exhibited strong hemolytic activity. In contrast, peptides V13S and L6A/L17A with less hydrophobicity showed weaker hemolytic activity. This is consistent with previous reports that hemolytic activity is correlated with hydrophobicity^2^,^15^,^16^. It is interesting that the hydrophobicity of V13E was stronger than peptide V13K due to the intramolecular salt bridge that stabilizes the α-helical structure. However, it showed less hemolytic activity which may be attributed to the charge repulsion of V13E and negatively charged phospholipids during the interaction of the peptide and membrane.

Antimicrobial activity of MAPs was determined by MIC. Two gram-positive bacteria (*S. aureus* and *S. epidermidis*) and two gram-negative bacteria (*P. aeruginosa* and *E. coli*) were used and the geometric mean MIC values of bacteria provided an overall evaluation of antimicrobial activity of MAPs against gram-negative or gram-positive bacteria, respectively. From Table 1, we can see that the MIC value of A12L/A20L is approximately 4 μM, and L6A/L17A is approximately 125 μM. The antimicrobial activity decreases along with the reducing hydrophobicity of the peptide. However, the antimicrobial activity of V13E is the weakest, despite having a higher hydrophobicity than V13S and V13K. This may be attributed to the negative charge of the glutamic acid residue, and the same trend can be seen in Table 2.

Four cancer cells (HeLa, A549, SGC7901, and B16) were used to evaluate the anticancer activity of MAPs and it is clear that the anticancer activity correlates with the hydrophobicity of the peptides (Table 2). Despite the cytotoxicity of MAPs against normal eukaryotic cells of 3T3-L1, they also exhibited the same trend. However, the IC_50_ values of anticancer activity of the peptides are less than the cytotoxicity of the peptides against normal cells. This indicates that peptides exhibited a strong specificity against cancer cell lines in this study.

### Interaction of peptides with liposome model membranes

**Figure 1:**
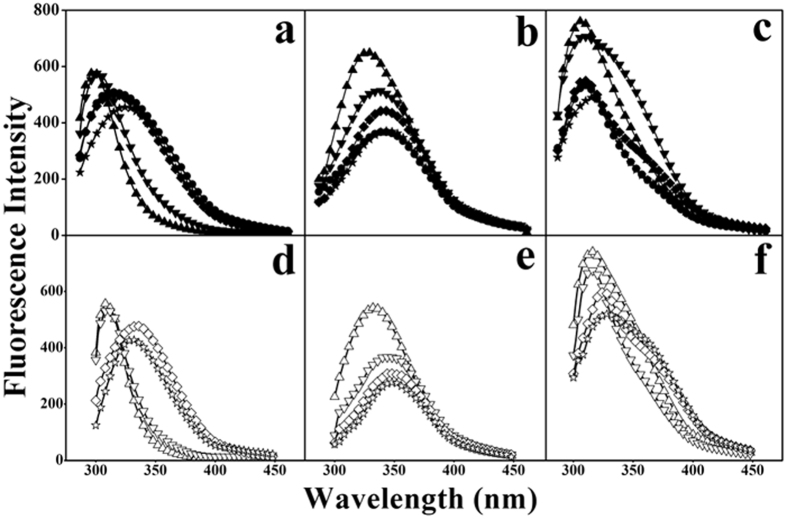
Tryptophan fluorescence emission spectra of MAPs with LUVs model membranes at 25 °C. PC/PG (7:3 w/w) LUVs were used to mimic normal eukaryotic cell membranes in Panel (**a**,**d**); PC/Chol (8:1 w/w) LUVs were used to mimic prokaryotic cell membranes in Panel (**b**,**e**); PC/SM/PE/PS/Chol (4.35:4.35:1:0.3:1 w/w) LUVs were used to mimic cancer cell membranes in Panel (**c**,**f**). Symbols used are as follows: ▲ for V13L; ▼ for V13A; ◆ for V13S; ● for V13K; ★ for V13E; △ for A12L/A20L; ▽ for A20L; ◊ for L6A; and ☆ for L6A/L17A.

In order to investigate the interaction between peptides and difference types of cell membranes, three different LUVs were used to mimic a prokaryotic cell membrane (PC/PG), a normal eukaryotic cell membrane (PC/Chol), and a cancer cell membrane (PC/SM/PE/PS/Chol). Tryptophan residue in the peptide sequence was used as a fluorescent probe to examine the interaction of peptides with model membranes. The fluorescence intensity of tryptophan and the blue shift in fluorescence wavelength would be significantly enhanced when peptides insert into a more hydrophobic environment^17^. The fluorescence emission maxima of MAPs is approximately 346–350 nm in the HEPES buffer, however, the fluorescence emission maxima of MAPs ranges from 309 nm to 350 nm and exhibits a significant blue shift when the peptides insert into the membrane mimicking environments (Fig. 1 and Supplementary Table S3). For example, in the PC/PG (7:3 w/w) anionic prokaryotic mimicking model membrane, peptides V13L and A12L/A20L had the strongest hydrophobicity with blue shift values of approximately 37 nm and 38 nm, respectively. Conversely, peptide V13E with a negative charge of glutamic acid residue, and peptide L6A/L17A with the lowest hydrophobicity had blue shift values of approximately 18 nm and 17 nm, respectively. These results are consistent with the MIC data, indicating that the blue shift values of peptides correlate with peptide hydrophobicity. In addition, within PC/SM/PE/PS/Chol (4.35:4.35:1:0.3:1 w/w) as a cancer-mimicking model membrane, the same results could be obtained in that peptide hydrophobicity correlates with anticancer activity and the blue shift values of peptides. However, compared with the negatively charged membranes, the blue shift values of peptides in the PC/Chol (8:1 w/w) zwitterionic eukaryotic mimicking model membrane were obviously reduced, i.e., the blue shift values of peptides V13L and A12LA20L were only 11 nm and 14 nm, respectively, and for others peptides the values ranged from 1 nm to 6 nm. Thus, these results indicate the low cytotoxicity of MAPs against normal cells, which are consistent with the MHC data.

### Single-molecule force spectroscopy

**Figure 2:**
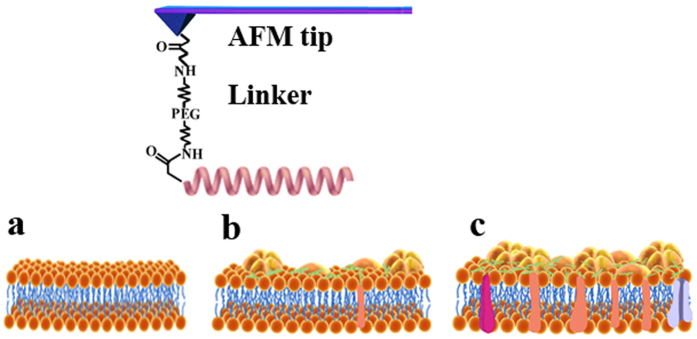
The schematic illustration of the peptide-functionalized AFM tip on different cell membranes. The peptide was covalently coupled to the AFM tip via a heterobifunctional PEG interact with LUVs mimicking membranes (**a**), *A. laidlawii* (**b**), and HeLa cells (**c**).

**Figure 3:**
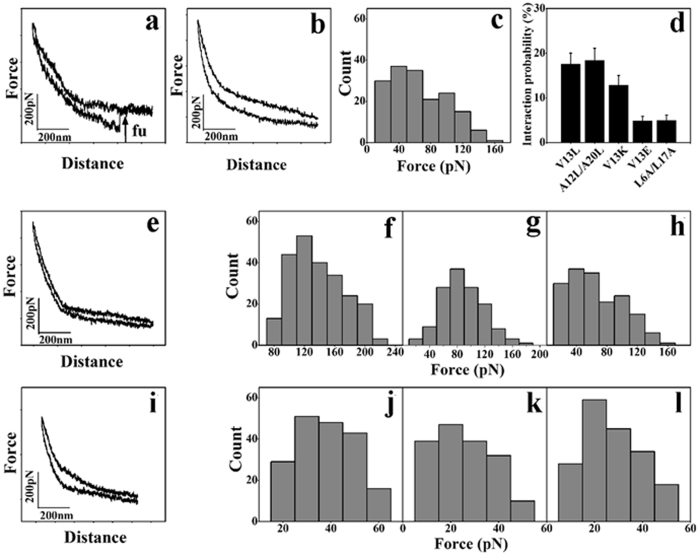
The force measurement of the interaction between single peptide V13L and different LUVs model membranes and biological cell membranes. Panel (**a)** denotes typical force curve presenting the interaction between V13L and chicken erythrocyte; Panel (**b)** denotes the interaction force curve of V13L interacting with chicken erythrocyte after blocking by Tris-HCl buffer; Panel (**c**) denotes the histogram of unbinding force between V13L and chicken erythrocyte; Panel (**d**) denotes the interaction probability of V13L with chicken erythrocyte; Panel (**e**) denotes the interaction force curve of V13L interaction with the PC/PG (7:3 w/w) after blocking by Tris-HCl buffer; Panel (**f–h**) denote the histogram of unbinding force between V13L and PC/PG (7:3 w/w), PC/Chol (8:1 w/w) and PC/SM/PE/PS/Chol (4.35:4.35:1:0.3:1 w/w), respectively; Panel (**i**) denotes the interaction force curve of V13L interaction with the *A. laidlawii* cells after blocking by Tris-HCl buffer; Panel (**j**–**l**) denotes the histogram of unbinding force between V13L and *A. laidlawii* cells, 3T3-L1 cells, HeLa cells, respectively.

Based on the above results, five selected peptides of V13L, A12L/A20L, V13K, V13E, and L6A/L17A, with different hydrophobicity and charges were used to examine the interaction probabilities and forces between peptides and membranes, both on the lipid model membranes and on the biological membranes by SMFS based on AFM, respectively. A cysteine residue was covalently introduced to the N-terminus of peptides and provided a free sulfhydryl group to modify the tips of the AFM. A PEG linker was used since each heterobifunctional PEG–NHS linker (approximately 20 nm) can only covalently connect with a single peptide molecule^18^, which guarantees a single-molecule interaction between MAPs and biological membranes in real time^19^. If an AFM tip was functionalized with two or more peptides, where several peaks were recorded. In the reports, data with two peaks were not collected and calculated. An interaction scheme between MAPs, the three model types, and biological membranes is shown in Fig. 2a–c. For one force–distance cycle, one peptide-functionalized AFM tip approached the model and biological membranes right on the top, and the subsequent tip was withdrawn from the cell membranes to retrace the process. The typical force curve of V13L against chicken erythrocyte membranes is shown in Fig. 3a. The arrow “fu” shows the force signal of V13L unbinding from the chicken erythrocyte membranes, and the interaction probability of the force signal is about 18% out of about 1000 force cycles (as shown in Fig. 3d). In addition, blocking experiment was used to test the specificity of these force events. As the electrostatic attraction is one important reason for peptides approaching model membranes and biological membranes, the Tris-HCl buffer including plenty amino groups^20^ was injected into the working chamber and inhibits the interactions between MAPs and model membranes/biological membranes. We found that most of the interaction force signals of peptides with model membranes and biological membranes disappeared in the force curves (Fig. 3b,e,i). The unbinding forces of V13L against different model membranes and biological membranes with the loading rate of 1 × 10^5^ pN s^−1^ are shown in Fig. 3c,f–h,j–l.

The unbinding forces of MAPs with three model membranes and four biological membranes are shown in Table 3. It is clear that on the uncharged membrane (PC/Chol), the values of the unbinding force of most MAPs are relatively small (approximately 100 pN). While on the two negatively charged phospholipids, such as the prokaryotic mimicking membrane and the cancer mimicking membrane, the values of the unbinding force are significantly higher (approximately 130 pN) than those in the PC/Chol. In addition, it is noteworthy that the values of the unbinding force of V13E are inevitably lower than other peptides with three model membranes owing to the extra negative charge. However, this is not the case with three biological membranes, indicating the different circumstances between artificial model membranes and real biological systems. Biological membranes are supermolecular structures that contain different types of lipids, proteins and polysaccharides^5^. It is interesting to see that there is not much difference among the values of the unbinding force of peptides with the three biological membranes (Table 3). The results are that V13K generally exhibited the largest force values among the peptide analogs in both the model and biological membranes, and the distribution of the unbinding force of V13K is also higher than other peptides. While the data of force events for MAPs interacting with chicken erythrocyte cells indicated that the unbinding forces of MAPs with chicken erythrocyte cells is smaller than those of peptides with model membranes, and bigger than those of peptides with biological membranes. The unbinding forces of V13E with chicken erythrocyte cells are remarkable lower than other peptides, which are similar with the results from three model membranes; and the unbinding force values of V13K with chicken erythrocyte cells are larger than others, which shows the same possibility as MAPs with other biological membranes. The results indicate that MAPs interaction with the chicken erythrocyte membranes including more lipid composition than other biological membranes had already bridged the gap between model membranes and other biological membranes.

**Figure 4:**
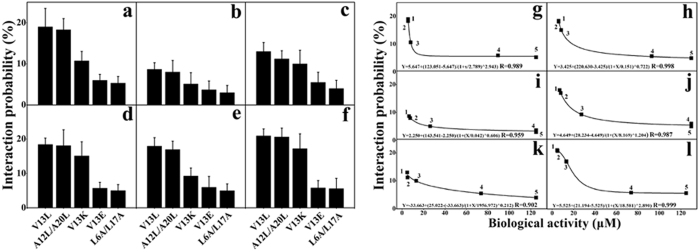
The interaction probability of MAPs with different LUVs model membranes and biological cell membranes, and relationships of interaction probability and biological activity. Panel (**a)** denotes the interaction probability of MAPs with PC/PG (7:3 w/w); Panel (**b)** denotes the interaction probability of MAPs with PC/Chol (8:1 w/w); Panel (**c)** denotes the interaction probability of MAPs with PC/SM/PE/PS/Chol (4.35:4.35:1:0.3:1 w/w); Panel (**d**) denotes the interaction probability of MAPs with *A. laidlawii* cells; Panel (**e**) denotes the interaction probability of MAPs with 3T3-L1 cells; and Panel (**f**) denotes the interaction probability of MAPs with HeLa cells. Panel (**g**) denotes the antibacterial activity (MIC) against gram-negative bacteria and the interaction probability on PC/PG (7:3 w/w). Panel (**h**) denotes IC_50_ values against 3T3-LI cells and the interaction probability on PC/Chol (8:1 w/w). Panel (**i**) denotes IC_50_ values against HeLa cells and the interaction probability on PC/SM/PE/PS/Chol (4.35:4.35:1:0.3:1 w/w). Panel (**j**) denotes the antibacterial activity (MIC) against gram-negative bacteria and the interaction probability on *A. laidlawii*. Panel (**k**) denotes IC_50_ values against 3T3-LI cells and the interaction probability on 3T3-L1. Panel (**l**) denotes IC_50_ values against HeLa cells and the interaction probability on HeLa cells. The peptides in this figure from left to right are V13L (1), A12L/A20L (2), V13K (3), V13E (4), and L6A/L17A (5). The experimental data are from Tables 1 and 2 and Fig. 4. All the correlation parameters of interaction probability and biological activity are >0.9.

The interaction probabilities between MAPs and different cell membranes were examined by AFM and the results are shown in Fig. 4a–f. The force signal of each peptide on model membranes and biological cell membranes of different LUVs was examined. For the model membranes, PC/PG (7:3 w/w), PC/Chol (8:1 w/w), and PC/SM/PE/PS/Chol (4.35:4.35:1:0.3:1 w/w) were used to mimic the prokaryotic cell, eukaryotic normal cell, and eukaryotic cancer cell, respectively (Fig. 4a–c). The interaction probability of peptides is quite different for different membranes. It is interesting to see that the values of interaction probability of all peptides on different mimicking model membranes are in the order of PC/PG > PC/SM/PE/PS/Chol > PC/Chol^9^. While for the living cells, *A. laidlawii,* 3T3-L1, and HeLa cells were used to represent prokaryotic cells, eukaryotic normal cells, and eukaryotic cancer cells, respectively (Fig. 4d–f). Although it is not as obvious as in model membranes, the values of interaction probability of all peptides on normal cells (3T3-L1 and chicken erythrocyte cells) are slightly lower than those on the other biological membranes. In addition, the values of interaction probability of MAPs generally correlate with peptide hydrophobicity, both on the three model membranes and on four biological membranes.

## Discussion

In this study, two groups of α-helical MAPs with different hydrophobicity were designed and utilized to systematically investigate the targeting specificity of MAPs on different types of cell membranes by SMFS and fluorescence spectroscopy. In previous studies, antimicrobial activity or anticancer activity of α-helical antimicrobial or anticancer peptides increased with the increase of peptide hydrophobicity^2^,^16^. In this study, peptide hydrophobicity and charge have been proven as key parameters for the interaction between MAPs and all tested model and biological membranes, which is the critical process affecting peptide biological activities including antimicrobial activity, anticancer activity, hemolytic activity, cytotoxicity against normal cells, and specificity (Tables 1 and 2). Except for the positively charged peptides exhibited stronger targeting interact with the bacterial and cancer cell membrane than the normal cell membrane.

Cell membrane permeabilization of MAPs against the outer and inner bacterial membranes was examined by NPN and ONPG experiments. From the results in Supplementary Fig. S2, it is clear that the membrane disruption abilities of MAPs against these bacterial membranes correlate with the hydrophobicity and net charge of MAPs, which is consistent with the antimicrobial activity of MAPs in Table 1. Thus, the results further indicate that bacterial membranes are the target of MAPs^21^. Through tryptophan fluorescence and quench experiments, MAPs exhibited strong targeting specificity against negatively charged model membranes (Fig. 1, Supplementary Table S3 and Fig. S3). That is, the positively charged peptides exhibited stronger insertion ability against the negatively charged model membrane than in the zwitterionic model membrane. The results indicate that the lipid composition may play an important role in the interactions between cell membranes and MAPs.

The unbinding force and interaction probability of MAPs with three types of model membranes and four types of biological membranes were determined by SMFS based on AFM (Figs 3 and 4a–f and Table 3). The electrostatic interaction between the positively charged peptides and the negatively charged model membranes plays an important role in the unbinding force, since the values of the unbinding force of peptides on the negatively charged LUVs were significantly greater than the uncharged LUVs (Table 3). The values of unbinding force of V13E on model membranes were smaller than other peptides, which may be attributed to the electrostatic repulsion between the negatively charged glutamic acid residue of V13E and the negatively charged PC/PG and PC/SM/PE/PS/Chol^22^. The results show that peptides targeting specifically against prokaryotic and cancer cell model membranes rather than normal eukaryotic cell model membranes. Compared with the model membranes data, the lower values of the unbinding force of peptides with three biological membranes indicate that the biological membranes are far more complicated than model membranes, which may be due to the proteins on cell membranes (Table 3). Because of the hydrophobicity and positive charges of peptides, the values of the unbinding force may be attributed to the negatively charged lipid more than the membrane protein, which results that the values of the unbinding force of peptides interacting with lipid model membranes are larger than those with biological membranes. It is interesting to see that V13K generally exhibited the largest force values among the peptide analogs in both the model and biological membranes, showing the importance of the combined effect of charge and hydrophobicity during the peptide–membrane interactions. While the value of the unbinding force of V13L and A12L/A20L was relatively small, despite the fact that they have a stronger hydrophobicity and biological activity, it shows that peptides with a stronger hydrophobicity would insert deeper into cell membranes and lose the electrostatic interaction with the polar head groups of phospholipids. This is consistent with the findings of a previous study where the force value variations of V13L and A12L/A20L were low, which shows that they are more consistent when interacting with membranes and may be more uniform in their sensitivity to different positions on membranes^19^. In contrast, the force value variations of other peptides were high, indicating that these peptides may remain in a less defined status when interacting with membranes and may be more variable in sensitivity to different positions on membranes^19^. Consistent with the biological activity and membrane permeability, the force values of V13E and L6A/L17A were lower than V13K, indicating the difficult insertion of these peptides into the hydrophobic core of membranes^19^,^23^. In addition, the interaction probability of MAPs with LUVs model membranes and biological cell membranes correlates with hydrophobicity and charge (Fig. 4a–f).

As shown in Fig. 4g–l, the five selected peptides of V13L, A12L/A20L, V13K, V13E, and L6A/L17A with different hydrophobicity and charges had strong correlations between the interaction probability on model and biological membranes and peptide biological activities. The fact that peptide biological activities exhibited similar correlations with the interaction probability of SMFS for both model and biological membranes indicates that the membrane is the sole target of MAPs. Single-molecule AFM has been proved as a sensitive and applicable technology to study peptide–membrane interactions. In addition, peptide hydrophobicity has been demonstrated to correlate with biological activities (Tables 1 and 2); hence, peptide antimicrobial and anticancer activities would be improved by the modulation of peptide hydrophobicity. It is interesting that the unbinding force and the interaction probability of V13E were smaller than other peptides, which may be attributed to the existence of the negatively charged glutamic acid residue which reduces the electrostatic interaction between the peptide and cell membranes.

**Figure 5:**
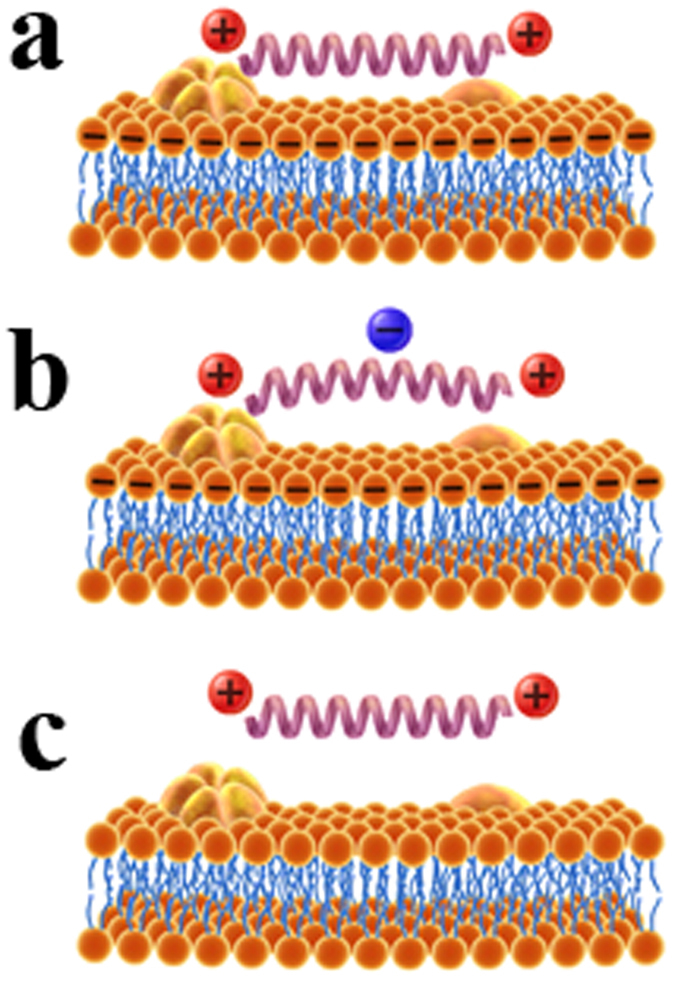
The model for the interaction mechanism between single peptide and different types of cell membranes. Panel (**a**) denotes peptide of V13K with positive charge interacting with the negatively charged lipid composition of membranes such as PC/PG (7:3 w/w) and PC/SM/PE/PS/Chol (4.35:4.35:1:0.3:1 w/w). Panel (**b**) denotes peptide of V13E with negative charged acid residue interaction with the negative charged lipid composition of membranes such as PC/PG (7:3 w/w) and PC/SM/PE/PS/Chol (4.35:4.35:1:0.3:1 w/w). Panel (**c**) denotes peptide of V13K with positive charge interaction with zwitterionic lipid composition of membranes such as PC/Chol (8:1 w/w).

It is well known that the initial driving force of peptides targeting microbes is the electrostatic interaction^23^. The facts that electrostatic forces are active over relatively long molecular distances and the lysine and arginine interactions with phosphate groups in lipid bilayers are particularly strong and contribute to the initial attraction and membrane-targeting step of many antimicrobial peptides^23^. Based on the above results, we illustrated MAPs specificity against different types of model membranes and biological membranes has been proved to be dependent on hydrophobicity, charge of peptides and the lipid composition of membranes. MAPs with positive charges would tend to interact with the negatively charged lipid composition of membranes as shown in Fig. 5a. Thus, the interaction force is mainly from the contribution of the electrostatic attraction. In contrast, MAPs with negatively charged acid residue interacting with the negatively charged lipid composition of membranes would produce electrostatic repulsion, which may reduce the force value as shown in Fig. 5b. Furthermore, MAPs with positive charges interacting with zwitterionic lipid composition of membranes would produce relatively lower force value for lacking the electrostatic attraction between peptides and lipid composition of membranes as shown in Fig. 5c. And the antimicrobial activity, anticancer activity and cytotoxicity against normal cells of MAPs are consistent with their abilities to interact with different biological membranes.

In this study, targeting specificity and mechanism of action of MAPs on different types of model membranes and biological membranes were systematically investigated by SMFS and spectrum technology. Peptide specificity against different types of membranes has been proved to be strongly dependent on the lipid composition of membranes. Positively charged MAPs showed strong targeting specificity against negatively charged membranes, such as prokaryotic and cancer cell membranes rather than normal eukaryotic cells, which is consistent with the “membrane discrimination” mechanism. Our results demonstrate that the selectivity of MAPs interaction with different cell membranes to improve targeting may be an applicable approach to design peptide therapeutics against bacterial resistance and cancer in clinical practices.

## Materials and Methods

All N-α-Fmoc-protected amino acids and rink amide 4-methylbenzhydrylamine resinwere purchased from GL Biochem (Shanghai, China). The test strains *E. coli* ATCC25922, *P. aeruginosa* ATCC27853, *S. aureus* ATCC25923, *S. epidermidis* ATCC12228, *E. coli* ML-35 ATCC43827, and *A. laidlawii* ATCC23206 were purchased from the American Type Culture Collection (Manassas, VA, USA). Mycoplasma broth base and mycoplasma selective supplement-G were purchased from OXOID (Beijing, China). Red blood cells (RBCs) used in the experiments were extracted from healthy blood donors. Human cervix carcinoma cells (HeLa), human lung carcinoma cells (A549), human gastric cancer cells (SGC7901), mouse melanoma cells (B16), and mouse fibroblasts (3T3-L1) were obtained from the American Type Culture Collection (Manassas, VA, USA). Chicken erythrocyte cells were drawn from wing vein. Chol, chicken egg PC, porcine brain PS, porcine brain PE, E. coli PG, and porcine brain SM were purchased from Avanti Polar Lipids, Inc. (Alabaster, AL, USA). AFM tips (Microlever) were purchased from Veeco Probes (Santa Barbara, CA, USA). All procedures were approved and supervised by the Animal Care and Use Committee, School of Life Sciences, Jilin University, (Changchun, Jilin, China). The entire procedure was carried out in accordance with related guidelines and regulations, and written informed consent was obtained from all subjects.

### Peptide synthesis and purification

All peptides were synthesized by solid-phase peptide synthesis using Fmoc (9-fluorenyl-methoxycar-bonyl) chemistry and rink amide 4-methylbenzhydrylamine resin (MBHA resin; 0.8 mmol/g), as described previously^2^. The crude peptides were purified on a LC-6A preparative reversed-phase high performance liquid chromatography (RP-HPLC, Shimadzu, Kyoto, Japan) using a Zorbax 300 SB-C8 column (250 × 9.4 mm inner diameter, 6.5 μM particle size, 300 Å pore size), as described previously^2^,^12^,^15^. The purity of the peptides was characterized by analytical RP-HPLC, mass spectrometry, and amino acid analysis.

### Circular dichroism (CD) analysis

The secondary structures of the peptides were determined using a Jasco J-810 CD spectrometer (Jasco, Easton, MD) at 25 °C 12. The benign buffer (pH 7.0, 100 mM KCl, 50 mM KH_2_PO_4_/K_2_HPO_4_) was used to simulate the hydrophilic environment and 50% 2,2,2-trifluoroethanol (TFE) (benign buffer: TFE = 1: 1, vol/vol) was used to simulate the hydrophobic environment. The mean residue molar ellipticities at 222 nm, [θ]_222_ (degree·cm^2^·dmol^−1^), were used to calculate the relative helical contents of MAPs^24^.

### Antimicrobial activity assay

Minimal inhibitory concentrations (MICs) were used to represent the antimicrobial activity of MAPs and were determined by the standard microtiter dilution method^12^. Four bacterial strains including two gram-negative bacterial strains of *E. coli* and *P. aeruginosa*, and two gram-positive bacterial strains of *S. aureus* and *S. epidermidis* were selected as representative prokaryotes^12^. A single bacterial colony was picked and cultured in Mueller–Hinton (MH) medium for 18–20 h. The bacterial solution was diluted in the same medium for a final inoculum of 5 × 10^5^ colony-forming units (CFU)/mL and placed in 96-well plates with 90 μL per well. Then, 10 μL of peptides was added. MICs were determined as the lowest peptide concentration that inhibited bacterial growth after incubation for 24 h at 37 °C. All assays were repeated in triplicate. GM is the geometric mean of MIC values from two gram-positive bacteria strains or two gram-negative bacteria strains in this table.

### Hemolytic activity assay

Peptide samples were serially diluted with phosphate-buffered saline (PBS) and added to 96-well plates (round bottom) with 70 μL per well. Healthy human red blood cells (hRBCs) were collected using an anticoagulation tube with EDTAK_2_ and centrifuged at 1,000 rpm for 5 min. The erythrocytes were washed three times, resuspended in PBS, and then diluted to a concentration of 2% in PBS. A total of 70 μL of 2% erythrocytes was added to each well and incubated at 37 °C for 2 h. The plates were centrifuged for 10 min at 3,000 rpm and the supernatant was transferred to a 96-well plate (flat bottom). The release of hemoglobin was determined by measuring the absorbance of the supernatant at 578 nm. The hemolytic activity was determined as the minimal peptide concentration that caused hemolysis [minimal hemolytic concentration (MHC)]. Erythrocytes in PBS and distilled water were used as the control of 0% and 100% hemolysis, respectively.

### Anticancer activity and cytotoxicity assay (IC_50_)

The MTT cell proliferation assay was used to investigate the anticancer activity and cytotoxicity of MAPs against cancer cell lines (HeLa, A549, SGC-7901, and B16) and normal cells (3T3-L1), respectively. Cells (5 × 10^3^) were seeded in 96-well plates and incubated overnight. The peptide samples were then added to cells with final concentrations ranging from 1 μM to 125 μM. After 24 h, 20 μL of 5 mg/mL MTT solution in PBS was added to the cells and treated for 4 h at 37 °C. The formazan crystals were dissolved by 150 μL of dimethyl sulfoxide (DMSO). Finally, the absorbance was determined at 492 nm. The results were expressed as IC_50_, representing the concentration at which cell viability was reduced by 50%. All assays were repeated in triplicate.

### Outer membrane permeability assay

The outer membrane permeabilization of MAPs against *E. coli* was evaluated using the hydrophobic fluorescent probe NPN (1-N-phenylnaphthylamine). *E. coli* was inoculated and cultured in LB medium for 18 h at 37 °C. Further, a 1 mL bacterial solution was inoculated into 50 mL of the LB medium and cultured at 37 °C until the absorbance at 600 nm was 0.4–0.6. After centrifugation (4,000 × *g* for 10 min), the bacteria were collected, resuspended in a buffer (pH 7.4, 5 mM HEPES, 5 mM NaN_3_), and adjusted to OD_600_ nm = 0.5. Thereafter, 300 μL of peptide samples was added to 2700 μL of resuspended bacterial solution for a final MAP concentration of 8 μM. The same volume of reaction buffer (pH 7.4, 5 mM HEPES, 5 mM NaN_3_) without peptides was prepared as the control. A Shimadzu RF-5301 PC fluorescent spectrometer (excitation wavelength of 350 nm, emission wavelength of 420 nm) was used to continuously collect data for 10 min^25^.

### Inner membrane permeability assay

The inner membrane permeabilization of MAPs against *E. coli* ML-35 was tested by the Shimadzu UV-2550 UV spectrophotometer. *E. coli* ML-35 was cultured in LB medium containing 5% lactose. The bacterial cells were collected and resuspended with sterile water and adjusted to OD_420_ nm = 1.2. A total of 1,000 μL of bacterial suspension was mixed with 100 μL of 30 mM o-nitrophenyl-β-D-galactosidase (ONPG), and then added to 900 μL of MAPs. The final peptide concentration was 8 μM. The negative control was prepared in a 0.5% NaCl solution^26^.

### Preparation of LUVs mimicking membranes

In accordance with previously described methods, LUVs were prepared with PC/PG (7:3 w/w), PC/Chol (8:1 w/w), and PC/SM/PE/PS/Chol (4.35:4.35:1:0.3:1 w/w) to mimic prokaryotic cell, cancer cell, and normal cell, membranes, respectively^11^,^12^. Lipids using the freeze-thaw method as described previously, followed by extrusion through 0.1 μM double-stacked nuclepore filters using an Mini-Extruder (Avanti Polar Lipids, Inc.)^11^,^27^.

### Tryptophan fluorescence and quenching assay

Three kinds of LUVs were prepared and cultured in HEPES buffer (pH 7.4, 10 mM HEPES, 150 mM NaCl) and the molar concentration was adjusted to 100 μM. A total of 14 μL of 100 μM MAPs was mixed with 686 μL of 100 μM liposomes at 25 °C for 10 min. A Shimadzu RF-5301PC, (excitation wavelength of 350 nm, emission wavelength of 300–450 nm) was used to measure the tryptophan fluorescence. Potassium iodide (KI) as a quencher was added to the previous reaction system for a final concentration of 0.02–0.08 M, respectively. The experimental data were plotted according to the Stern–Volmer equation F_0_/F = 1 + Ksv[Q], where F_0_ and F are the fluorescence intensity in the absence or presence of a quencher at concentration [Q], respectively, and Ksv is the Stern–Volmer quenching constant^28^.

### AFM assay

The AFM tip was first washed with concentrated sulfuric acid and hydrogen peroxide (3:1, v:v). The tip was dried using ozone (O_3_) for 15 min and dried overnight in a dryer. The tip was then modified with APTES under an argon atmosphere and the peptide connected with a PEG–NHS linker^19^. Finally, the AFM tip linked with the peptide was washed three times in PBS and kept at 4 °C. A 500-μL LUV suspension was deposited on Lys coated glass and the LUVs were absorbed on the surface for 1 h at 65 °C 9,29. *A. laidlawii* was deposited on poly-L-lysine coated glass and absorbed for 1 h at 37 °C 30. The 3T3-L1 and HeLa cells were cultured on glass slides in Dulbecco’s modified Eagle’s medium (DMEM). Before performing force spectroscopy experiments, the cells were washed with fresh DMEM three times to remove the cell metabolites^19^. Isolation of chicken erythrocytes was carried out at room temperature. Then the erythrocytes were washed three times in 2 mL PBS buffer by centrifuge (1,000 rpm) and resuspended in PBS buffer. 200 μL erythrocyte suspension were deposited onto poly-L-lysine coated glass for 20 min^10^. The force measurements of MAPs with LUVs mimicking membranes and living cells were performed using the AFM5500 (Agilent Technologies, Chandler, AZ, USA)^19^.

## Additional Information

**How to cite this article**: Sun, S. *et al.* Specificity and mechanism of action of alpha-helical membrane-active peptides interacting with model and biological membranes by single-molecule force spectroscopy. *Sci. Rep.* **6**, 29145; doi: 10.1038/srep29145 (2016).

## Supplementary Material

Supplementary Information

## Figures and Tables

**Table 1:** MIC and MHC of peptides against bacteria and hRBCs.

Peptides	MHC (μM)	G^+^ MIC (μM)			G^−^ MIC (μM)		
		*S. aureus*ATCC25923	*S. epidermidisb*ATCC12228	GM	*P. aeruginosa*ATCC27853	*E. coli*ATCC25922	GM
V13L	4	8	2	4	8	4	5.66
V13A	16	8	2	4	8	4	5.66
V13S	125	8	4	5.66	8	8	8
V13K	250	32	4	11.31	8	8	8
V13E	>500	125	125	125	125	64	89.44
A12L/A20L	4	8	2	4	8	4	5.66
A20L	32	8	2	4	8	4	5.66
L6A	500	>125	64	126.49	125	64	89.44
L6A/L17A	>500	125	125	125	125	125	125

**Table 2:** IC_50_ of peptides against cancer cell lines and normal cells.

Peptides	IC_50_ (μM)				
	HeLa	A549	SGC7901	B16	3T3-L1
V13L	4.86	2.80	2.00	3.51	6.55
V13A	5.52	6.83	2.33	3.62	6.79
V13S	12.18	12.07	4.27	5.61	17
V13K	13.12	24.36	18.94	11.93	26.21
V13E	73.66	109.68	92.04	120.44	>125
A12L/A20L	5.11	4.66	2.96	2.85	7.55
A20L	6.62	8.27	6.62	10.45	13.9
L6A	94.67	92.39	78.87	74.65	87.3
L6A/L17A	>125	>125	>125	>125	>125

**Table 3:** Root mean square (RMS ± SD) distribution of the unbinding force values for peptide analogs with all types of membranes.

Peptides	PC/PG (pN)	PC/Chol (pN)	PC/SM/PE/PS/Chol (pN)	*A. laidlawii*(pN)	3T3-L1 (pN)	HeLa (pN)	Chicken erythrocyte (pN)
V13L	127 ± 34	98 ± 30	124 ± 40	27 ± 9	30 ± 11	31 ± 10	76 ± 34
V13K	135 ± 36	101 ± 37	128 ± 51	51 ± 18	51 ± 15	52 ± 20	87 ± 39
V13E	111 ± 37	89 ± 34	92 ± 34	55 ± 12	51 ± 11	36 ± 14	59 ± 35
A12L/A20L	128 ± 38	99 ± 40	122 ± 44	37 ± 8	37 ± 7	32 ± 10	71 ± 36
L6A/L17A	132 ± 41	100 ± 34	130 ± 36	41 ± 15	39 ± 12	39 ± 11	78 ± 39
