# Supplementary material for: Specificity and mechanism of action of alpha-helical membrane-active peptides interacting with model and biological membranes by single-molecule force spectroscopy
Source: Sci Rep. 2016 Jul 1;6:29145. doi: 10.1038/srep29145 (PMC4929710; doi:10.1038/srep29145)
Supplement: Supplementary Information [file srep29145-s1.doc]

Specificity and mechanism of action of alpha-helical membrane-active peptides interacting with model and biological membranes by single-molecule force spectroscopy

Shiyu Sun^a, c^, Guangxu Zhao^a, c^, Yibing Huang^a, b, c^, Mingjun Cai^d^, Yuping Shan^d^, Hongda Wang^d,e*^, Yuxin Chen^a, b, c^*

^a^Key Laboratory for Molecular Enzymology and Engineering of the Ministry of Education, Jilin University, Changchun, P.R. China 130012

^b^National Engineering Laboratory for AIDS Vaccine, Jilin University, Changchun, P.R. China 130012

^c^School of Life Sciences, Jilin University, Changchun, P.R. China 130012

^d^State Key Laboratory of Electroanalytical Chemistry, Changchun Institute of Applied Chemistry, Chinese Academy of Sciences, Changchun, P.R. China 130022

^e^University of Chinese Academy of Sciences, Beijing 100049, P.R. China.

**Running title**: Specificity and mechanism of action of membrane-active peptides

**Corresponding Authors:** Yuxin Chen, Key Laboratory for Molecular Enzymology and Engineering of the Ministry of Education, Jilin University, 2699 Qianjin St., Changchun, Jilin, P. R. China 130012. Tel.: +86-431-85155220 Fax: +86-431-85155200 Email: chen_yuxin@jlu.edu.cn; Hongda Wang, State Key Laboratory of Electroanalytical Chemistry, Changchun Institute of Applied Chemistry, Chinese Academy of Sciences, Changchun, Jilin, P. R. China. 130022. Tel.: +86-431-85262684, Fax: +86-431-85262864, Email: hdwang@ciac.ac.cn.

**Key words:** membrane-active peptides, specificity, mechanism of action, atomic force microscopy, membranes

**Peptide design**

Based on the previous studies, the hydrophobicity of peptide plays a key role for the activity of antimicrobial and anticancer peptides and could be affected by two ways, changing the intrinsic hydrophobicity of side chains by substituting amino acids or altering the number of the *i*→*i*+3 and *i*→*i*+4 hydrophobic interactions, which affects the continuity of the hydrophobic face of the peptide. Based on this principle, in this study, a 26-residue amphipathic MAP of V13K,[^1^](#_ENREF_1) which exhibited strong antimicrobial activity and anticancer activity, was used as a framework to design two groups of peptide analogs, the hydrophobicity of peptides was systematically changed and used to investigate the interactions of peptides with different types of cell membranes by AFM. The sequences and hydrophobicity of MAPs used in this study are shown in Table S1. L-amino acids are expressed with one-letter codes; bold italic letters represent the substitute of different hydrophobic amino acid residues，and *t*_R_ means the retention time during RP-HPLC of all peptides.

In one group, leucine, alanine, serine and glutamic acid were used to replace lysine, which in the central site of the hydrophobic face of parent peptide V13K to obtain four peptide analogs of V13L, V13A,V13S and V13E with different side-chain hydrophobicity, respectively. In this study, glutamic acid residue as a negatively charged amino acid was used to form V13E and to study the effect of net charge on the interaction between peptide and cell membrane. In the other group, leucine or alanine on the nonpolar face of the peptide was used to substitute the original alanine or leucine with the single or double substitutions to obtain four peptide analogs of A12L/A20L, A20L, L6A, L6A/L17A with different numbers of the *i*→*i*+3 and *i*→*i*+4 hydrophobic interactions.[^3^](#_ENREF_3) Thus, alanine residue was replaced by leucine residue to increase peptide hydrophobicity and leucine residue was substituted by alanine residue to decrease the hydrophobicity of the peptide. As shown in Table 1, the hydrophobicity of peptide was represented by retention time of RP-HPLC (*t_R_*), which is in the order of V13L>V13A>V13E>V13S>V13K and A12L/A20L>A20L >V13K>L6A>L6A/L17A.

**Peptide secondary structure**

CD spectroscopy illustrated the secondary structure of peptide analogs under benign environment and 50% TFE to mimic the hydrophobic conditions of cell membranes. CD results are shown in Supplementary Table S2 and Fig. S1. It is clear that the secondary structure of MAPs with double minima at 208 nm and 222 nm in 50% TFE, indicating that peptides can be induced to a α-helical structure in the hydrophobic environment and A12L/A20L exhibited the highest helical structure among all the analogs in 222 nm. The helical content (%) of peptides in two groups relative to the molar ellipticity value of the peptide A12L/A20L in 50% TFE. In addition, except the peptide of V13E, the relative helicity of the peptides are in the same order of V13L>V13A>V13S>V13K and A12L/A20L>A20L>L6A>L6A/L17A in the benign and hydrophobic environments and also in the same order with the peptide hydrophobicity.

**Cell membrane permeabilization**

Gram-negative bacterial membranes are composed of inner membrane and outer membrane. MAPs interact with both bacterial membranes to exhibit their antimicrobial activity.In this study, NPN was used as a hydrophobic fluorescent probe to study the disruption ability of MAPs against the outer membrane of *E. coli*. In a hydrophilic environment, the intensity of NPN is weak and the fluorescence intensity gradually increase when NPN transfer into a hydrophobic environment due to the damage of bacterial outer membrane by MAPs.[^4^](#_ENREF_4) Compared to the control, the fluorescence intensity of NPN significantly enhanced in a short time after treated with MAPs, particularly for the peptides with high hydrophobicity, such as V13L and A12L/A20L (Supplementary Fig. S2a and S2b), which indicating that peptide hydrophobicity plays an important role in the outer membrane permeabilization against Gram-negative bacteria.

Furthermore，the disruption ability of MAPs against the inner membrane of bacteria also was examined using lactose permease deficient bacteria *E. coli* ML-35 by ONPG experiment and the results are shown in Supplementary Fig. S2c and S2d. ONPG is lactose analog and can be hydrolyzed by the β- galactosidase in cytoplasmic matrix and transformed into galactose and o-nitrophenol in yellow color. Thus, o-nitrophenol levels could be detected by UV spectrum at 420 nm and evaluated the disruption ability of MAPs against the inner membrane of bacteria.[^5^](#_ENREF_5) It is clear that the fluorescence intensities of peptides were significantly enhanced in 10 min, indicating that MAPs damaged the inner membrane of bacteria quickly. In the similar trend to the NPN experiment, V13L and A12L/A20L exhibited the higher fluorescence intensity than other peptides in the ONPG experiment, showing to the importance of peptide hydrophobicity in disrupting the inner membrane of bacteria. Again, charge of peptide also plays a crucial role in both inner and outer membrane permeabilization since the permeability of V13E was weaker than that of V13K.

**Quenching Experiments**

A water soluble KI quencher was used to quench the tryptophan fluorescence of peptides in the solution since tryptophan fluorescence would not be quenched when the peptides entered into the hydrophobic LUVs.[^6^](#_ENREF_6) KI quenching effect curves are shown in Supplementary Fig. S3 and the quenching curve slope represents the quenching constant. It is clear that the degrees of KI quenching of peptides with high hydrophobicity (V13L and A12L/A20L) are very weak. However, the degrees of KI quenching of MAPs with low hydrophobicity (V13E and L6A/L17A) are relatively strong, since it is hard for them to enter into the hydrophobic core of a membrane. These results also showed that peptides with high hydrophobicity interacted strongly with the membranes. In addition, MAPs also exhibited targeting specificity against negatively charged model membranes, such as PC/PG (7:3 w/w) and PC/SM/PE/PS/Chol (4.35:4.35:1:0.3:1 w/w), due to the electrostatic interactions between the positively charged MAPs and the negatively charged membranes (see Supplementary Fig. S3).

**References**

1．Chen, Y. *et al.* Rational design of alpha-helical antimicrobial peptides with enhanced activities and specificity/therapeutic index. *J. Biol. Chem.* **280**, 12316-12329 (2005).

2．Chen, Y. *et al.* Role of peptide hydrophobicity in the mechanism of action of alpha-helical antimicrobial peptides. *Antimicrob. Agents Chemother.* **51**, 1398-1406 (2007).

3. Huang, Y. B., Wang, X. F., Wang, H. Y., Liu, Y. & Chen, Y. Studies on mechanism of action of anticancer peptides by modulation of hydrophobicity within a defined structural framework. *Mol. Cancer Ther.* **10**, 416-426 (2011).

4. Loh, B., Grant, C. & Hancock, R. E. Use of the fluorescent probe 1-N-phenylnaphthylamine to study the interactions of aminoglycoside antibiotics with the outer membrane of Pseudomonas aeruginosa. *Antimicrob. Agents Chemother.* **26**, 546-551 (1984).

5. Je, J. Y. & Kim, S. K. Chitosan derivatives killed bacteria by disrupting the outer and inner membrane. *J. Agric. Food Chem.* **54**, 6629-6633 (2006).

6. Huang, Y. *et al.* Role of helicity of alpha-helical antimicrobial peptides to improve specificity. *Protein & cell* **5**, 631-642 (2014).


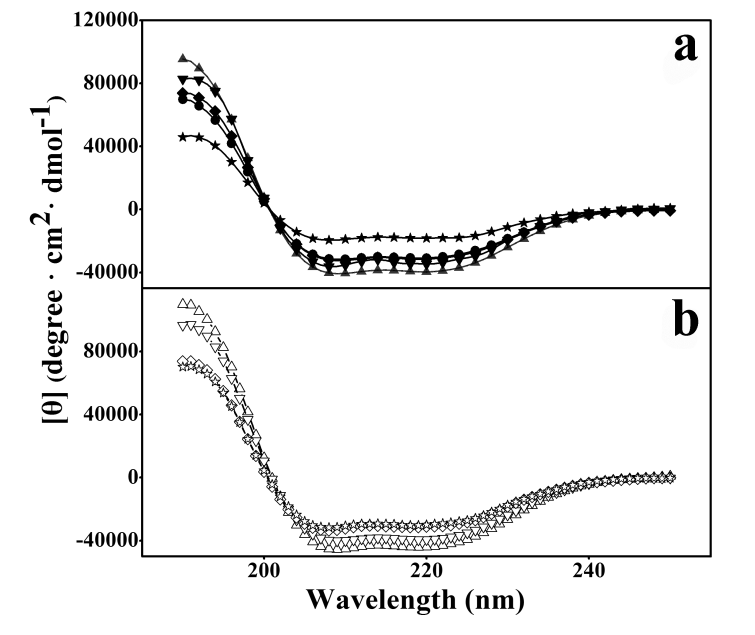


**Figure S1. CD spectra of peptide analogs.** Panels a and b denote the CD spectra of peptides in the KP buffer and 50% TFE. Symbols used are as follows: ▲ for V13L; ▼ for V13A; ◆ for V13S; ● for V13K; ★ for V13E; △ for A12L/A20L; ▽ for A20L; ◇ for L6A; and ☆ for L6A/L17A.


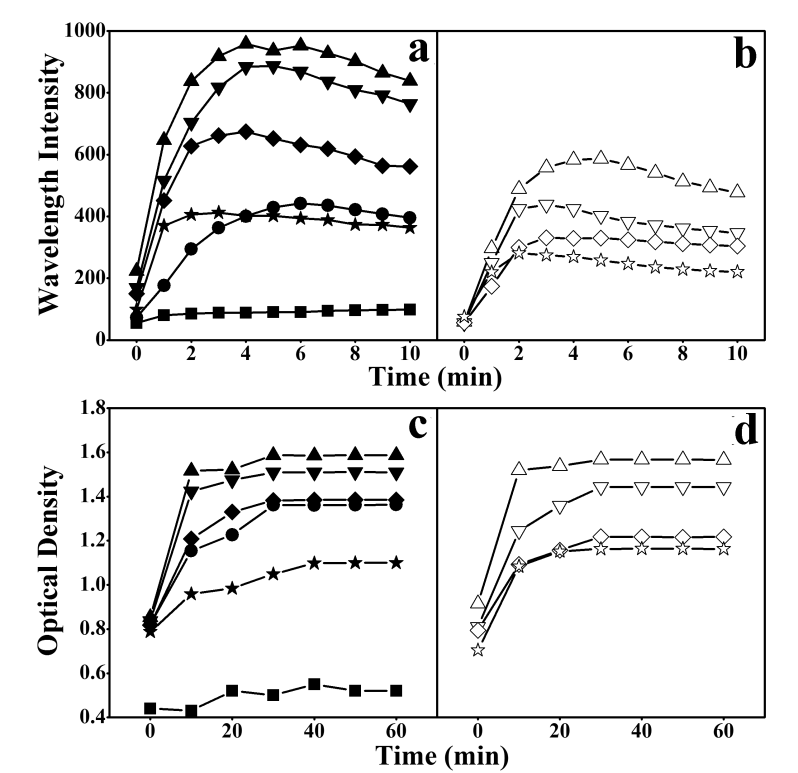


**Figure S2. Membrane permeabilization assay of MAPs.** Panels a and b, outer membrane permeabilization induced by MAPs was detected by NPN uptake in *E.coli*. Panels c and d, the effect of MAPs on the inner membrane permeabilization of *E. coli* ML-35. Release of cytoplasmic β-galactosidase activity (measured from the absorbance at OD_420 nm_) from *E. coli* ML-35 treated by MAPs. Symbol used are as follows: ▲ for V13L; ▼ for V13A; ◆ for V13S; ● for V13K; ★ for V13E; △ for A12L/A20L; ▽ for A20L; ◇ for L6A; ☆ for L6A/L17A, and ■ for Control.


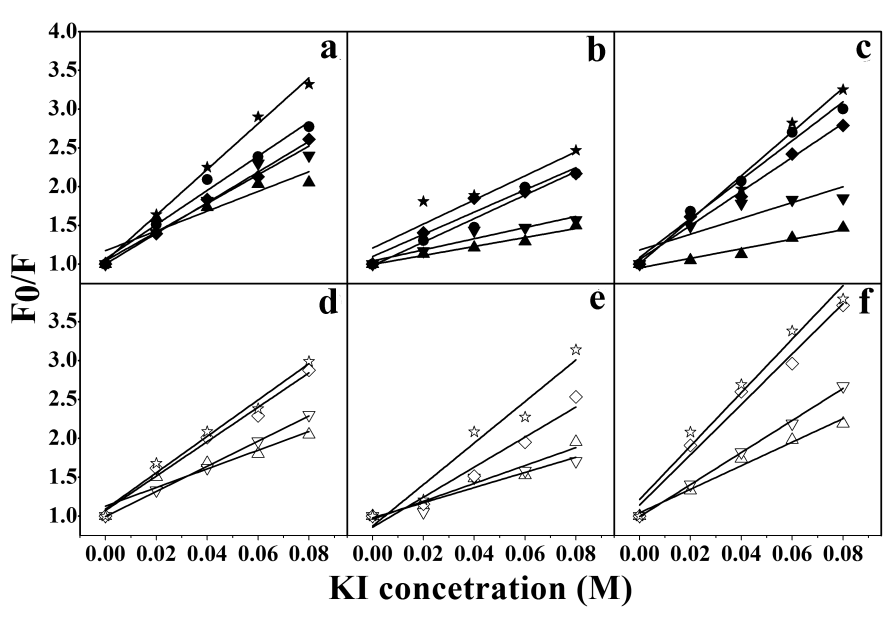


**Figure S3. Stern–Volmer plots of MAPs with various LUVs models at 25°C. Stern–Volmer plots were obtained by the sequential addition of the fluorescence quencher KI.** PC/PG (7:3 w/w) LUVs were used to mimic normal eukaryotic cell membranes in Panel a and Panel d; PC/Chol (8:1 w/w) LUVs were used to mimic prokaryotic cell membranes in Panel b and Panel e; PC/SM/PE/PS/Chol (4.35:4.35:1:0.3:1 w/w) LUVs were used to mimic cancer cell membranes in Panel c and Panel f. Symbols used are as follows: ▲ for V13L; ▼ for V13A; ◆ for V13S; ● for V13K; ★ for V13E; △ for A12L/A20L; ▽ for A20L; ◇ for L6A; and ☆ for L6A/L17A.

| Peptides | | Amino acid sequence | | *t*_R_ (min) |
| --- | --- | --- | --- | --- |
| V13L | Ac-KWKSFLKTFKSA***L***KTVLHTALKAISS-amide | | 53.177 | |
| V13A | Ac-KWKSFLKTFKSA***A***KTVLHTALKAISS-amide | | 47.337 | |
| V13S | Ac-KWKSFLKTFKSA***S***KTVLHTALKAISS-amide | | 43.038 | |
| V13K | Ac-KWKSFLKTFKSAKKTVLHTALKAISS-amide | | 38.790 | |
| V13E | Ac-KWKSFLKTFKSA**E**KTVLHTALKAISS-amide | | 44.290 | |
|  |  | |  | |
| A12L/A20L | Ac-KWKSFLKTFKS***L***KKTVLHT***L***LKAISS-amide | | 47.457 | |
| A20L | Ac-KWKSFLKTFKSAKKTVLHT***L***LKAISS-amide | | 44.019 | |
| L6A | Ac-KWKSF***A***KTFKSAKKTVLHTALKAISS-amide | | 36.053 | |
| L6A/L17A | Ac-KWKSF***A***KTFKSAKKTV***A***HTALKAISS-amide | | 32.210 | |

**Table S1 Sequences and RP-HPLC retention times of α-helical peptides used in this study**

| Peptides | Benign | | 50% TFE | |
| --- | --- | --- | --- | --- |
|  | [θ]_222_ | α-Helix (%) | [θ]_222_ | α-Helix (%) |
| V13L | −10502.62 | 24.24 % | −39315.30 | 90.73% |
| V13A | −6524.79 | 15.06 % | −34527.20 | 79.68% |
| V13S | −4400.44 | 10.15 % | −30899.20 | 71.30% |
| V13K | −4236.54 | 9.77 % | −30268.40 | 69.85% |
| V13E | −3876.86 | 8.95 % | −18689.59 | 43.13% |
|  |  |  |  |  |
| A12L/A20L | −16812.70 | 38.80 % | −43334.10 | 100.00% |
| A20L | −8809.41 | 20.33 % | −38820.90 | 89.59% |
| L6A | −3836.18 | 8.85 % | −30626.20 | 70.67% |
| L6A/L17A | −3589.82 | 8.28 % | −28421.80 | 65.58% |

**Table S2 CD data of peptides**

| Peptides | HEPES (nm) | PC/PG | | | PC/Chol | | | PC/SM/PE/PS/Chol | | |
| --- | --- | --- | --- | --- | --- | --- | --- | --- | --- | --- |
|  |  | Wavelength  (nm) | Blue shift (nm) | Intensity | Wavelength  (nm) | Blue shift  (nm) | Intensity | Wavelength  (nm) | Blueshift  (nm) | Intensity |
| V13L | 346 | 309 | 37 | 226.71 | 335 | 11 | 281.14 | 316 | 32 | 380.24 |
| V13A | 347 | 313 | 34 | 239.11 | 343 | 4 | 173.85 | 316 | 29 | 368.92 |
| V13S | 350 | 323 | 27 | 286.74 | 347 | 3 | 85.51 | 319 | 29 | 342.76 |
| V13K | 348 | 320 | 28 | 221.68 | 344 | 4 | 78.00 | 321 | 27 | 247.36 |
| V13E | 349 | 331 | 18 | 223.81 | 348 | 1 | 136.25 | 326 | 23 | 260.90 |
|  |  |  |  |  |  |  |  |  |  |  |
| A12LA20L | 348 | 310 | 38 | 315.46 | 334 | 14 | 302.46 | 316 | 32 | 393.97 |
| A20L | 349 | 310 | 39 | 263.08 | 343 | 6 | 141.39 | 317 | 32 | 335.48 |
| L6A | 349 | 330 | 19 | 193.64 | 348 | 1 | 64.43 | 326 | 23 | 295.77 |
| L6AL17A | 350 | 334 | 17 | 236.28 | 350 | 0 | 71.60 | 329 | 21 | 213.77 |

**Table S3 Tryptophan fluorescence emission maxima and intensity of peptides in HEPES buffer or in the presence of three model membranes**
